# Visible through touch: open-source 3D-printed tools for inclusive microbiology education

**DOI:** 10.1099/acmi.0.001179.v3

**Published:** 2026-05-21

**Authors:** Claire L. Price, Aidan Seeley, Hayley Pincott, Daniel J. Morse, Llinos G. Harris, J. Gareth Noble

**Affiliations:** 1 Swansea University Medical School, Singleton Park Campus, Swansea SA2 8PP, UK; 2 SUMS3D, Swansea University Medical School, Singleton Park Campus, Swansea SA2 8PP, UK; 3 See Science, Cardiff CF10 3DD, UK; 4 School of Cellular and Molecular Medicine, University of Bristol, Bristol BS8 1TD, UK; 5 Faculty of Social and Life Sciences, Wrexham University, Wrexham LL11 2AW, UK

**Keywords:** 3D printing, accessibility, inclusive education, microbiology teaching, open-source tools, tactile learning

## Abstract

Microbiology education relies heavily on visual interpretation of microbe cultures, microscopy and assays, which can create barriers for blind and visually impaired learners. Inclusive teaching requires multimodal tactile approaches that make microbiology accessible to all students. This work presents two open-source browser-based web applications (the Agar Plate STL Generator and Microscope Slide to STL) designed to convert standard microbiological images into 3D-printable tactile models. Both tools use heightmap-based image processing to translate visual data into topographic 3D surfaces. Users can upload digital images of agar plates or microscope slides (e.g. in jpg format), adjust relief depth through an interactive interface and generate printable STL files without prior experience in computer-aided design or 3D printing. The resulting tactile models replicate spatial and morphological features of microbial growth and microscopy, enabling learners to ‘see through touch’. These tools enable independent participation by visually impaired students and promote multi-sensory engagement for all learners. Both applications and their source code are freely available under a Creative Commons Attribution–NonCommercial 4.0 licence via GitHub and Zenodo. Open-source tactile microbiology tools provide a scalable, inclusive means to bridge the accessibility gap in STEM education. By converting visual laboratory outputs into tangible models, these resources support universal design for learning and foster equitable participation in microbiology.

**Impact Statement**

Microbiology is traditionally a highly visual science, dependent on observing (for example, visualizing colony morphologies, colour changes and microscopic structures). This reliance on sight creates an accessibility gap for blind and visually impaired learners, who are often excluded from practical engagement in laboratory-based teaching. The tools presented here (the Agar Plate STL Generator and Microscope Slide to STL) provide an open-source practical solution to this long-standing challenge by transforming visual microbiological data into tactile 3D models. By enabling students to explore microbial structures and growth patterns through touch, these resources empower visually impaired learners to participate independently and equitably in microbiology education. These tactile models can benefit all learners by encouraging multi-sensory engagement, improving spatial understanding and supporting neurodiverse learning preferences. As freely available browser-based tools, they can be readily adopted by educators without specialist technical expertise, promoting inclusivity across diverse educational settings. This work demonstrates how open-source technology can be leveraged to make microbiology ‘visible through touch’, fostering a more inclusive and engaging learning environment for the next generation of microbiologists.

## Data Summary

The authors confirm all supporting data, code and protocols have been provided within the article or through supplementary data files. All files for the tools mentioned can be downloaded from GitHub or Zenodo. They are licensed under the Creative Commons Attribution–NonCommercial 4.0 International Licence. Agar Plate STL Generator – https://github.com/claireprice/Agar-Plate-STL-Generator; https://zenodo.org/records/15682097. Microscope Slide to STL – https://github.com/claireprice/microscope-to-stl/tree/v1.0.0; https://zenodo.org/records/15706644.

## Introduction

### The accessibility gap in microbiology education

Microbiology, like many STEM fields, relies heavily on visual information, from observing colony morphologies on agar plates to interpreting microscope images and colour-changing assays. This dependence on sight poses a significant accessibility gap for learners who are blind or partially sighted. Globally, an estimated 36 million people live with blindness (including ~1 million children) and over 200 million have moderate to severe visual impairments [1]. Yet, traditional science education often offers few alternatives to its visual teaching methods [1]. In a typical microbiology laboratory, for example, students are expected to *see* bacterial/fungal colonies, *read* colour indicators or *view* cells under a microscope. These are tasks that exclude those with limited vision. This gap not only hinders participation of blind and visually impaired students but also underscores a broader need for inclusive design in science education.

Importantly, making microbiology more accessible benefits more than just visually impaired learners. Embracing multi-sensory tactile learning can aid a wide range of students, including those who are neurodivergent or who learn better through hands-on interaction. It has been shown that incorporating movement and touch in learning can improve memory and cognitive function [2,3]. Providing information in multiple modes (visual, tactile, auditory) aligns with Universal Design for Learning principles (https://udlguidelines.cast.org/), ensuring that education is adaptable to diverse needs. In short, tactile inclusive design helps level the playing field [4]. It allows all learners to engage with microbiological concepts in meaningful ways. By closing the accessibility gap, not only do we welcome underrepresented students into the laboratory but also enrich the learning experience for everyone through multi-sensory engagement.

### Tactile learning in microbiology: why it matters

Tactile learning offers an alternate ‘window’ into the microbial world for those who cannot rely on sight. Many microbiological phenomena are inherently spatial or structural, i.e. the shape of a microbial cell, the zones of inhibition around an antibiotic disc and the pattern of colonies on agar. These are typically conveyed through images or direct observation. However, with tactile models, these phenomena can be made ‘visible’ through touch. For example, a student with vision loss might feel a raised outline of streaked colonies on a model agar plate or feel the image being shown under a microscope. Access to these types of resources allows blind and visually impaired students to engage with the same material in real time [5,6]. Such tactile models transform abstract visuals into concrete objects that can be explored by hand. Colonies become bumps; growth patterns become textures.

This is a powerful approach as it promotes inclusive collaboration. When blind and sighted learners share the same tactile model, each can interpret it through their strongest sense and reach understanding together. For instance, translucent 3D prints called lithophanes can be used to produce tactile x-rays. Held against a light source, a lithophane reveals a picture for sighted viewers, while its embossed surface conveys the same information through touch [7]. The key is that tactile tools do not segregate the learning experience; they foster a shared understanding accessible to all participants.

Tactile learning can also support students with other needs. Neurodivergent learners may find that handling physical models helps maintain focus or aids comprehension by providing concrete references. Tactile input can reduce cognitive load for some learners by externalizing information (quite literally in this case, as the concept can be held in the student’s hands). Educators have observed that simple adaptations like raised-line diagrams, textured maps or 3D models can make science more inclusive [5,6].

Importantly, 3D-printed objects enhance learning for *all* students, not just those with specific accessibility needs. They bring a tangible, interactive dimension to abstract scientific ideas, allowing learners to physically engage with concepts such as microbial morphology, cell structure or growth dynamics. This multi-sensory engagement can deepen understanding, spark curiosity and improve retention across diverse learning profiles [8]. In microbiology, a tactile approach might involve feeling the relative size difference between a bacterium and a yeast cell via scaled 3D models or tracing the outline of a microbial growth curve converted into a raised-line graph. These strategies align with a growing movement to diversify how science is taught, embracing neurodiversity and ensuring that students who struggle with traditional visual or text-based materials have alternative pathways to understanding.

Tactile inclusive design matters in microbiology education because it breaks down barriers imposed by vision-centric teaching. It enables blind and visually impaired students to actively participate in laboratory sessions and lectures, turning microbiology into a multi-sensory science. It also enriches the pedagogical toolkit for instructors, offering new ways to illustrate concepts (e.g. ‘feel this colony morphology’) that can benefit all learners. As we strive to train the next generation of microbiologists, such inclusivity is not just about compliance or sympathy, it is about unleashing talent and curiosity that might otherwise be left untapped in the dark.

## Technical overview of two novel tactile tool web apps

Here we present two browser-based web apps that we have created that will aid the development of tactile microbiology 3D-printed tools without specialized knowledge of computer-aided design software or 3D printing on the part of the user. These web apps allow users to produce tactile 3D-printed agar plates or microscope slides from an uploaded image.

### The Agar Plate STL Generator

**Fig. 1.**
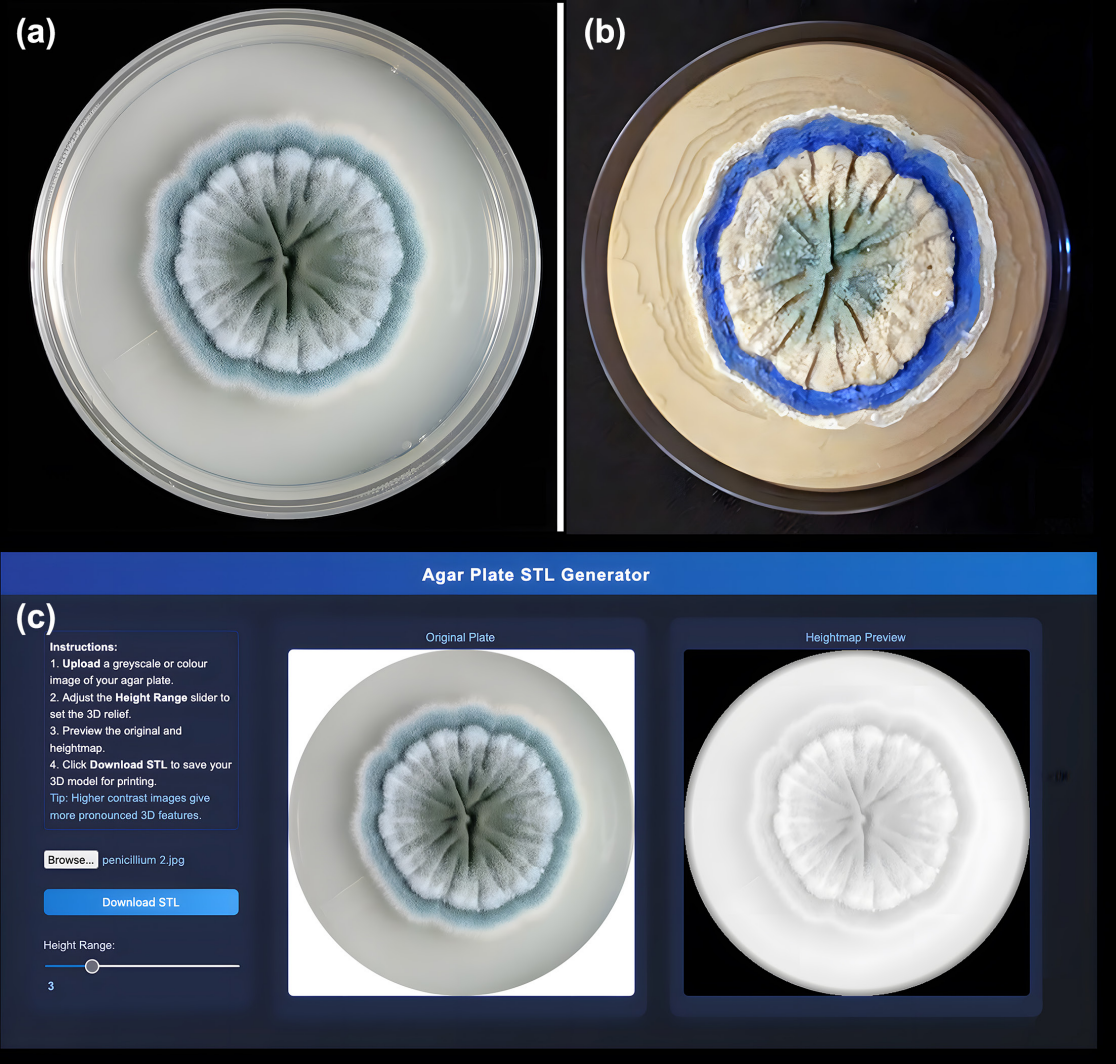
Generation of a 3D-printable tactile agar plate using the Agar Plate STL Generator. (**a**) Photograph of a *Penicillium* sp. colony growing on agar, showing characteristic radial morphology and blue-green pigmentation. (**b**) Corresponding 3D-printed tactile model produced using the Agar Plate STL Generator, with raised features representing colony growth and texture. (**c**) Screenshot of the web application interface displaying the uploaded image (left) and the generated heightmap preview (right) used to create the STL file. The tool converts pixel intensity into height to produce a topographic model suitable for 3D printing.

The Agar Plate STL Generator (https://claireprice.github.io/Agar-Plate-STL-Generator/) is a software tool that is a free-to-use, browser-based web app that converts images of agar plates into 3D models for suitable printing [9]. The goal is to allow users to take any photograph or digital image of an agar plate (for instance, a Petri dish growing bacterial colonies or fungal cultures) and generate a tactile model of that plate. By producing a 3D-printed replica where raised surfaces represent colonies or other features, the tool makes it possible to feel what was originally only visible (Fig. 1).

The Agar Plate STL Generator works by using the concept of a heightmap. It reads the brightness or colour intensity of each pixel in the input image and translates it into height in a 3D surface. Areas of the image that are lighter (or darker, depending on settings) will become taller in the 3D model, and areas that are uniform will form a flat region. This results in a topographic representation of the agar plate content. For example, imagine a photograph of a red agar plate with white fungal colonies; the colonies are ‘seen’ as brighter spots on the image. The generator will map those spots, transforming them into raised bumps on the model, while the agar background (darker by contrast) stays lower. The user of the tool can adjust the scaling of the heights using a slider, which changes how pronounced the 3D relief becomes (Fig. 1) [9]. This ensures that even subtle details (like faint microbial growth or slight colour zones) can be exaggerated in height until they are easily discernible by touch.

### Microscope Slide to STL

The Microscope Slide to STL web app (https://github.com/claireprice/microscope-to-stl) is a free, browser-based software tool that converts images of microscope slides into a file format suitable for 3D printing [10].

**Fig. 2.**
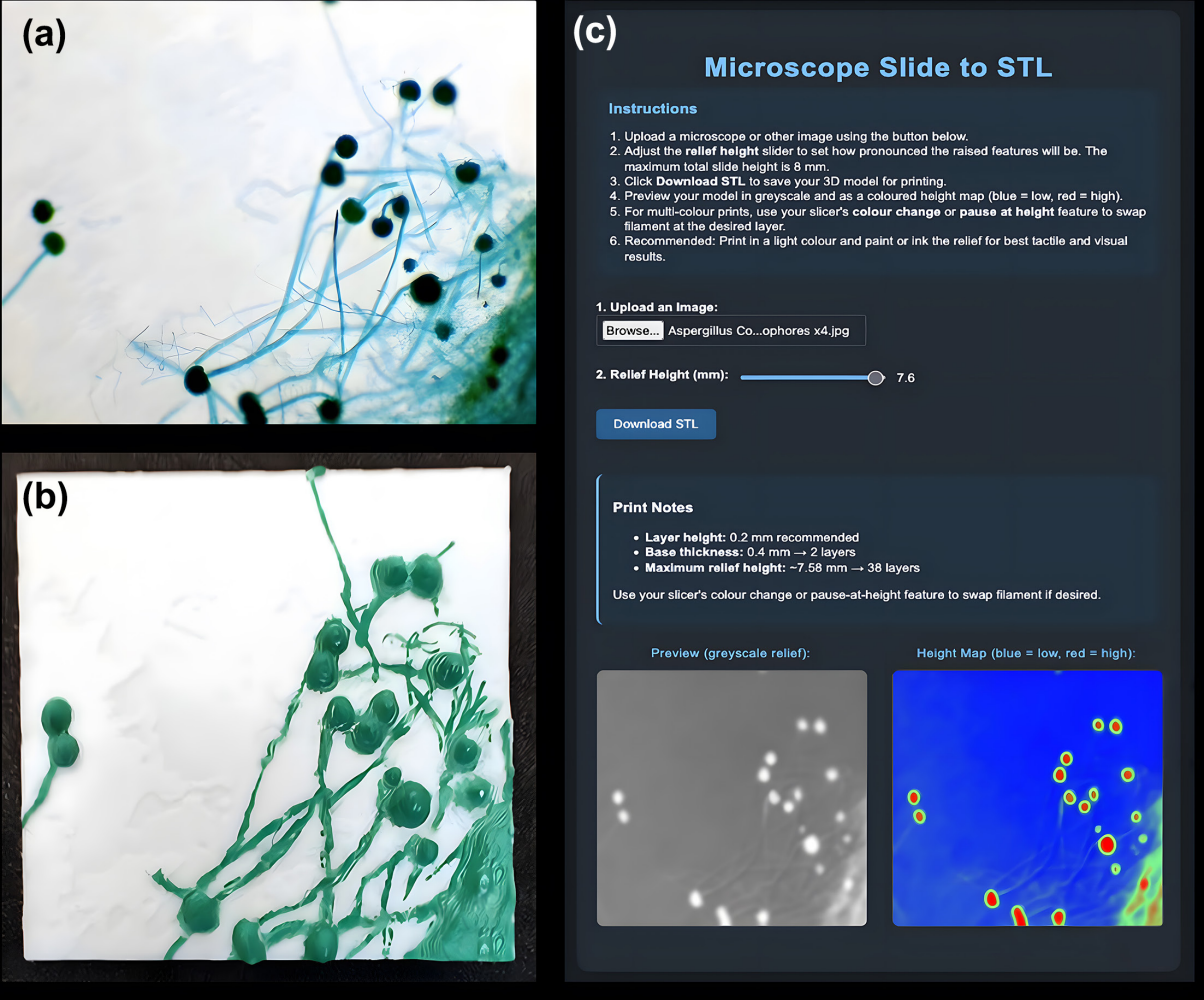
Generation of a 3D-printable tactile microscope slide using the Microscope Slide to STL web application. (**a**) Light micrograph of *Aspergillus* sp. showing characteristic conidiophores and spore heads. (**b**) Corresponding 3D-printed tactile model produced using the Microscope Slide to STL tool, with raised surfaces representing hyphae and spore-bearing structures. (**c**) Screenshot of the web application interface displaying the uploaded image (top), adjustable relief height slider and heightmap previews (bottom). The software converts image intensity into height data, producing a topographic 3D representation suitable for printing and tactile exploration.

Similar to the Agar Plate STL Generator, the Microscope Slide to STL web app uses a heightmap to make the image tactile. Thus, allowing the 2D microscope image to be represented in 3D. The user is able to adjust the thickness and, in turn, the difference in the overall 3D relief of the resulting 3D print by adjusting the Relief Height slider (Fig. 2). Utilizing a heightmap allows this concept to become a reality, resulting in a topographic representation of the microscope slide image (Fig. 2). The resulting model can be resized to suit the needs of the students/instructor.

### Producing multicoloured 3D prints

Multicoloured 3D prints can be produced using several approaches, depending on printer capability and the level of detail required. Where dual- or multi-extruder 3D printers (specifically fused deposition modelling printers) can be used to assign different coloured filaments to distinct regions of the model during slicing, enabling colouring of the model during printing. For single-extruder systems, multicolour 3D prints can be produced by introducing programmed pauses at defined layer heights within the slicing software. This allows the operator to manually change filament to create the colour transitions. In all cases, a multicoloured 3D print can be achieved by using acrylic paint in the post-processing process. This can allow for fine details to be added and can reduce the need for multiple filaments (this technique was used to produce Fig. 1b).

### Example potential use case – implementation in an inclusive microbiology teaching session

The Microscope Slide to STL web app could be used to support the teaching and assessment of c.f.u. estimation and microscopic cell counting. The tool could be used to produce tactile 3D models with a defined number of cells and used alongside standard teaching materials. These 3D models would allow students to physically explore cell distribution, identify individual cells and distinguish between isolated cells and clustered structures.

This approach could address a common challenge in microbiology teaching, where it is difficult to verify whether students are accurately identifying and counting cells during microscopy-based exercises. By providing models derived from images with known cell counts, educators could offer a controlled and reproducible way to access these skills. Students could practise counting and estimation techniques with reference to a known outcome, supporting both learning and feedback. It may also reduce dependency on oversubscribed teaching laboratories and allow laboratory-based skills to be learnt anywhere.

Importantly, this method could also enable visually impaired learners to develop and practise c.f.u.-related skills that are traditionally dependent on visual microscopy. By accessing the same information through touch (rather than sight), these students could engage more independently in cell identification and counting tasks. In addition, tactile models may reinforce understanding for sighted learners by linking abstract counting methods to a tangible representation of microbial populations.

This use case highlights how tactile tools could support both inclusivity and improved confidence in core quantitative microbiology skills.

### Licensing and usage

#### Licensing

Both web tools are released under a permissive Creative Commons License (CC BY-NC 4.0) so that others can freely use and adapt the code [9]. The source code and documentation are publicly available on GitHub (https://github.com/claireprice/Agar-Plate-STL-Generator and https://github.com/claireprice/microscope-to-stl, respectively), and an archived copy with DOI is provided via Zenodo for citation (https://zenodo.org/records/15682097 and https://zenodo.org/records/15706644, respectively).

#### Usage

Whilst these are predominantly web-based tools, they are cross-platform and can be run offline (simply by opening the respective index.html file in a browser), an intentional design choice to make it usable even in classroom or laboratory environments without internet access. Technical users can modify the code to add features or tailor it to specific needs (for instance, a braille label could be added to 3D output to mark the sample name or date). Since the tools are image-based, it is quite versatile. Any visual pattern on an agar plate (whether colonies, plaques or even hand-drawn markings) or microscope slide can be turned into a tactile model. This flexibility means the web apps could be applied not just for vision-impaired accessibility but also for creative science communication (e.g. making ‘microbial art’ lithophanes) or for creating durable 3D records of microbial experiments.

### Tactile tools enhance learning

The primary aim of tools like these is to support microbiology learning by creating an alternative sensory pathway. By converting visual laboratory results into physical objects, these tools enable students to literally get a feel for microbiological concepts. This approach can be transformative in several ways. By introducing a tactile dimension to information that is typically interpreted visually, it encourages deeper engagement and can support a more meaningful understanding of microbiological concepts. It also contributes to a more inclusive learning environment, particularly for students with visual impairments, by offering an alternative way to access and interpret laboratory data. In addition, physically interacting with these 3D models can make abstract ideas feel more concrete, which may enhance both student engagement and the retention of knowledge over time.

For example, microbiology often deals with spatial patterns (such as the arrangement of colonies on a plate, the clustering of cells in a biofilm, the clear zone around an antibiotic disc). A tactile model preserves spatial relationships, allowing blind or visually impaired learners to grasp patterns that would otherwise require sight. For example, the classic concept of isolating single colonies in a streak plate can be taught by having students feel a 3D print of a successful streak plate versus an overgrown one. The difference between isolated bumps and a confluent smear is immediately apparent to the touch.

Additionally, for all students, touching a 3D model can reinforce learning [8]. It turns an abstract 2D image into a concrete 3D experience. This multi-sensory engagement can improve recall and understanding. A student might both see and feel a microscope image of budding yeast, encoding the concept in multiple modalities. Such redundancy is known to aid learning, especially for students who might have difficulties with one mode of representation. Tactile models invite interaction and curiosity. For instance, students can pass around a safe *Mycobacterium tuberculosis*, sparking discussion and questions that might not arise from a textbook picture.

Perhaps most importantly, tactile tools allow students with disabilities to participate more independently and confidently. Rather than relying entirely on a lab partner’s commentary (which puts the student in a passive role), a blind or visually impaired student with a tactile model in hand can make their own observations. This fosters a sense of agency and inclusion. It also signals to the class that accessibility is valued; an outcome that benefits classroom culture. The shared use of tactile models by all students can destigmatize accommodations. When an entire class enjoys examining a 3D-printed microbial community, the tools are seen as part of innovative teaching rather than a special provision for one person.

It should be noted that making imagery tactile can sometimes reveal nuances that even sighted observers might miss. For instance, slight differences in elevation on a printed model could correspond to varying colony thickness or media depth that were not obvious in the flat image. In this way, tactile models might complement visual analysis. They encourage learners to observe (or feel) systematically and notice details.

These tools serve a dual role: teaching core concepts and raising awareness about accessibility and the different ways people perceive the world. In essence, tactile tools can enhance learning not only by filling an accessibility gap but also by enriching the pedagogical experience for all.

### Conclusion

Open-source 3D-printable labware and tactile teaching tools represent a promising and practical approach to make microbiology more accessible. By harnessing 3D-printing technology and the spirit of collaboration, educators can turn visual lab content into touchable experiences, inviting blind and low-vision learners (as well as others who benefit from hands-on learning) to fully participate in the exploration of the microbial world. The Agar Plate STL Generator and Microscope Slide to STL web app are examples of how a simple idea (converting plate/slide images to raised models) can be realized and shared openly to address an educational need.

Making microbiology ‘visible through touch’ is more than a technical exercise; it is a statement of inclusion. It says that microbiology is for everyone, regardless of how they perceive the world. Through open-source, 3D-printed tactile tools, we can begin to close the accessibility gap and inspire a diverse new generation of microbiologists who literally and figuratively grasp the concepts we teach. The petri dish and the microscope, the symbols of discovery in microbiology, need not be objects seen only by the eyes; they can be objects felt in the hands, their stories read by fingers, their science accessible to all.
